# Aminotriazole Alleviates Acetaminophen Poisoning via Downregulating P450 2E1 and Suppressing Inflammation

**DOI:** 10.1371/journal.pone.0122781

**Published:** 2015-04-17

**Authors:** Yuping Jing, Kunwei Wu, Jiashuo Liu, Qing Ai, Pu Ge, Jie Dai, Rong Jiang, Dan Zhou, Qian Che, Jingyuan Wan, Li Zhang

**Affiliations:** 1 Department of Pathophysiology, Chongqing Medical University, Chongqing, China; 2 Department of Physiology, Chongqing Medical University, Chongqing, China; 3 Hospital of Chongqing University of Arts and Sciences, Chongqing, China; 4 Laboratory of Stem cell and Tissue Engineering, Chongqing Medical University, Chongqing, China; 5 Department of Pharmacology, Chongqing Medical University, Chongqing, China; University of Missouri-Kansas City, UNITED STATES

## Abstract

Aminotriazole (ATZ) is commonly used as a catalase (CAT) inhibitor. We previously found ATZ attenuated oxidative liver injury, but the underlying mechanisms remain unknown. Acetaminophen (APAP) overdose frequently induces life-threatening oxidative hepatitis. In the present study, the potential hepatoprotective effects of ATZ on oxidative liver injury and the underlying mechanisms were further investigated in a mouse model with APAP poisoning. The experimental data indicated that pretreatment with ATZ dose- and time-dependently suppressed the elevation of plasma aminotransferases in APAP exposed mice, these effects were accompanied with alleviated histological abnormality and improved survival rate of APAP-challenged mice. In mice exposed to APAP, ATZ pretreatment decreased the CAT activities, hydrogen peroxide (H_2_O_2_) levels, malondialdehyde (MDA) contents, myeloperoxidase (MPO) levels in liver and reduced TNF-α levels in plasma. Pretreatment with ATZ also downregulated APAP-induced cytochrome P450 2E1 (CYP2E1) expression and JNK phosphorylation. In addition, posttreatment with ATZ after APAP challenge decreased the levels of plasma aminotransferases and increased the survival rate of experimental animals. Posttreatment with ATZ had no effects on CYP2E1 expression or JNK phosphorylation, but it significantly decreased the levels of plasma TNF-α. Our data indicated that the LD_50_ of ATZ in mice was 5367.4 mg/kg body weight, which is much higher than the therapeutic dose of ATZ in the present study. These data suggested that ATZ might be effective and safe in protect mice against APAP-induced hepatotoxicity, the beneficial effects might resulted from downregulation of CYP2E1 and inhibiton of inflammation.

## Introduction

Acetaminophen (N-acetyl-p-aminophenol, APAP) is the most widely used non-prescription analgesic and antipyretic drug throughout the world [1]. It is usually safe when used at therapeutic doses, but acute overdoses of APAP could cause severe and fatal hepatotoxicity [2–3]. APAP-induced hepatotoxicity is the leading cause of acute liver failure in the developed countries [2, 4]. In hepatocytes, the cytochrome P450, mainly CYP2E1, mediates the metabolism of APAP and leads to the generation of a highly reactive metabolite N-acetyl-p-benzoquinoneimine (NAPQI) [5–6]. The hepatotoxicity of APAP largely depends on NAPQI, which leads to severe oxidative liver injury [7].

The endogenous antioxidant enzymes such as catalase (CAT) play important defensive roles and provide protective benefits in oxidative stress [8]. Deficiency of CAT in acatalasemic mice or in the presence of aminotriazole (ATZ), a commonly-used CAT inhibitor [9–10], usually resulted in enhanced oxidative injury [11–14]. However, it was recently found that the CAT inhibitor ATZ significantly attenuated lipopolysaccharide (LPS)-induced acute lung injury in mice [15]. Consistent with this finding, Our recent study also found that treatment with ATZ attenuated carbon tetrachloride (CCl_4_)-induced acute liver injury [16]. Because CCl_4_ caused more severe liver damage in acatalasemic mice [17], the protective effects of ATZ in CCl_4_ poisoning could hardly result from of CAT inhibition. Therefore, ATZ might be a hepatoprotective reagent in oxidative liver injury but the underlying mechanisms remain unknown.

Because CCl_4_ poisoning is not common in clinical patients but APAP overdose frequently induces life-threatening hepatotoxicity, the potential hepatoprotective effects of ATZ on oxidative liver injury and the underlying mechanisms were further investigated in a mouse model with APAP-induced hepatotoxicity, a commonly used model mimicking clinical settings [18–19]. The efficiency of ATZ on hepatotoxicity was determined by aminotransferases measurement, histopathological examination and survival analysis. In addition, because the hepatotoxicity of APAP largely depends on CYP2E1-mediated metabolism of APAP and the activation of c-jun-N-terminal kinase (JNK) [20–21], the potential effects of ATZ on CYP2E1 and JNK were also investigated. Finally, the safety of these pharmacological interventions was evaluated via determination of the LD_50_ of ATZ in mice.

## Materials and Methods

### Animals

Six-week-old male C57 mice weighing 20–25 g were obtained from the Experimental Animal Center of Chongqing Medical University. The animals were given a standard laboratory diet and water ad libitum. All mice were maintained under specific pathogen-free conditions at a temperature of 20–25°C, 50±5% relative humidity under a 12 h dark/light cycle. The animals were acclimatized for at least l week before use. All experimental procedures involving animals were approved by the Animal Care and Use Committee of Chongqing Medical University.

### Reagents

ATZ, APAP and glutathione (GSH) assay kit were produced by Sigma (St. Louis, MO, USA). The kits for detection of alanine aminotransferase (ALT), aspartate aminotransferase (AST), myeloperoxidase (MPO), hydrogen peroxide (H_2_O_2_) and the kits for CAT assay were purchased from Nanjing Jiancheng Bioengineering Institute (Nanjing, China). The malondialdehyde (MDA) detection kits were purchased from Beyotime Institute of Biotechnology (Jiangsu, China). The enzyme-linked immunosorbent assay (ELISA) kits for detecting mouse tumor necrosis factor-alpha (TNF-α) were purchased from NeoBioscience Technology Company (Shenzhen, China). The rabbit anti-mouse JNK, phosphorylated JNK (p-JNK), CYP2E1 and β-actin antibodies were purchased from Abcam (Cambridge, UK). The BCA protein assay kit, horseradish peroxidase-conjugated goat anti-rabbit antibody and enhanced chemiluminescence (ECL) reagents were obtained from Pierce Biotechnology (Rockford, IL).

### Experimental protocol

Fresh suspensions of APAP were prepared before each experiment by dissolving the compound in warm phosphate buffered saline (PBS, pH 7.2). Acute liver injury was induced in mice by intraperitoneal (i.p.) injection of APAP (350 mg/kg). To evaluate the prophylactic effects of ATZ, vehicle or various dose of ATZ (125 mg/kg, 250 mg/kg or 500 mg/kg, dissolved in normal saline, i.p., the volume of ATZ solutions in each of experimental groups was 5 ml/kg) was administered 0.5 h prior to APAP challenge (n = 8 per group). The mice were then returned to their cages and provided food and water ad libitum. The animals were sacrificed by decapitation at 8 h after APAP exposure, blood samples were harvested for detecting TNF-α and measuring aminotransferases. The right lobe of the liver was fixed in formalin for morphological analysis. Remaining liver tissues were thoroughly washed in cold physiological saline and stored at -80°C until required. To evaluate the therapeutic effects, ATZ (500 mg/kg, i.p.) was administered at 1 h, 2 h or 4 h after APAP challenge, blood and liver samples were harvested at 8 h after APAP insult (n = 8 per group).

### Survival analysis

To determine the prophylactic effect of ATZ on mortality of APAP-challenged mice, vehicle or various dose of ATZ (125 mg/kg, 250 mg/kg or 500 mg/kg) was administered 0.5 h prior to APAP challenge (n = 20 per group); to determine the therapeutic effects of post-treatment with ATZ, ATZ (500 mg/kg) was administered at 1 h, 2 h or 4 h after APAP challenge (n = 20 per group). The survival rate after APAP administration was assessed four times a day for at least 7 days and the cumulative survival curve was depicted using the Kaplan-Meier method.

### Determination of the median lethal dose (LD_50_) of ATZ

To assess the safety of ATZ dose, the LD_50_ of ATZ was determined. Based on the data from our pretests, the mice were randomly assigned into four groups with 20 animals in each group and they were injected intraperitoneally with a series of doses of ATZ (5200 mg/kg, 5460 mg/kg, 5733 mg/kg, and 6019 mg/kg). The percentage survival was recorded in each group for at least 7 days. The LD_50_ and 95% confidence limit were calculated by the probit analysis method.

### Aminotransferases determination

Liver injury was assessed at 8 h after APAP administration by measuring plasma enzyme activities of ALT and AST using the detection kits according to the manufacturer's instructions.

### Histological analysis

Formalin-fixed specimens were embedded in paraffin and stained with hematoxylin & eosin routinely for conventional morphological evaluation under light microscope (Olympus, Tokyo, Japan). The degree of hepatocellular necrosis and hemorrhage were semi-quantified using a 0 (no lesion) to 4 (severe change) scoring system in 20 random fields at 100× magnification per animal (n = 3 per group) by a blinded pathologist as previously described [22].

### TNF-α determination

Plasma samples were harvested and the concentrations of TNF-α were determined using ELISA kits according to the manufacturer’s instructions.

### MPO activity assay

The activity of MPO was determined with MPO assay kit according to the manufacturer’s instructions. Briefly, the frozen liver tissues were thawed and homogenized in phosphate buffer containing 0.5% hexadecyltrimethylammonium bromide. The enzyme activity was determined by the MPO detection kit according to the manufacturer’s instructions. The MPO activity was assessed according to the absorbance measured at 450 nm. All values were normalized by the total protein concentration of each sample.

### CAT activity assay

The activity of CAT was determined with CAT assay kit according to the manufacturer’s instructions. Briefly, samples were treated with excess H_2_O_2_ for decomposition by CAT for 5 min, and the remaining H_2_O_2_ coupled with a substrate was treated with peroxidase to generate a red product absorbs maximally at 520 nm. CAT activity was thus determined by measuring the decomposed H_2_O_2_. All values were normalized by the total protein concentration of each sample.

### H_2_O_2_ determination

The content of H_2_O_2_ in liver tissue was determined with H_2_O_2_ assay kit according to the manufacturer’s instructions. H_2_O_2_ could oxidize Fe^2+^ to Fe^3+^, and then Fe^3+^ reacted with xylenol orange leading to colorimetric reaction that could be further detected by a spectrometer at a wavelength of 560 nm. The concentration of H_2_O_2_ was calculated according to standard concentration curve and normalized by the total protein concentration of each sample.

### GSH measurement

The the content of reduced glutathione in liver tissues was determined with glutathione assay kit according to the manufacturer’s instructions. The concentration of GSH was assessed according to the absorbance measured at 412 nm and calculated according to standard concentration curve. All values were normalized by the total protein concentration of each sample.

### MDA measurement

Liver tissues were prepared to make 1:10 (w/v) homogenates, and the homogenates were then centrifuged at 12,000 g under 4°C for 20 min to collect supernatants for determination of MDA. The concentrations MDA was evaluated by the thiobarbituric acid-reactive substances method (TBARS) using a MDA detection kit according to the manufacturer's instructions. All values were normalized by the total protein concentration of each sample.

### Western blot analysis

Total proteins from frozen liver samples were prepared according to the method described by the protein extract kit (Beyotime, Shanghai, China). The total protein concentration was determined using the BCA protein assay kit (Pierce, USA). Protein extracts were fractionated on 12% polyacrylamide-sodium dodecyl sulfate (SDS) gel and then transferred to nitrocellulose membrane. The membrane was blocked with 5%(w/v) nonfat milk in Tris-buffered saline (TBS) containing 0.05% tween-20, and then the membrane was incubated with primary antibody overnight at 4°C, followed by incubation with secondary antibody. Antibody binding was visualized with an ECL chemiluminescence system (Pierce, USA) and short exposure of the membrane to X-ray films (Kodak, Japan).

### Statistical analysis

All data except the survival rate were expressed as a mean ± SD. Multigroup comparisons were performed using one-way ANOVA with the Turkey's post hoc test. The statistical significance between two groups was determined by the *Student's t* test. The statistical significance of the semi-quantified scores was determined by the Wilcoxon rank sum test. The survival statistics were compared with a Kaplan-Meier curve and log-rank test. Results were considered statistically significant when *P*<0.05.

## Results

### Pretreatment with ATZ alleviated APAP-induced hepatotoxicity

**Fig 1:**
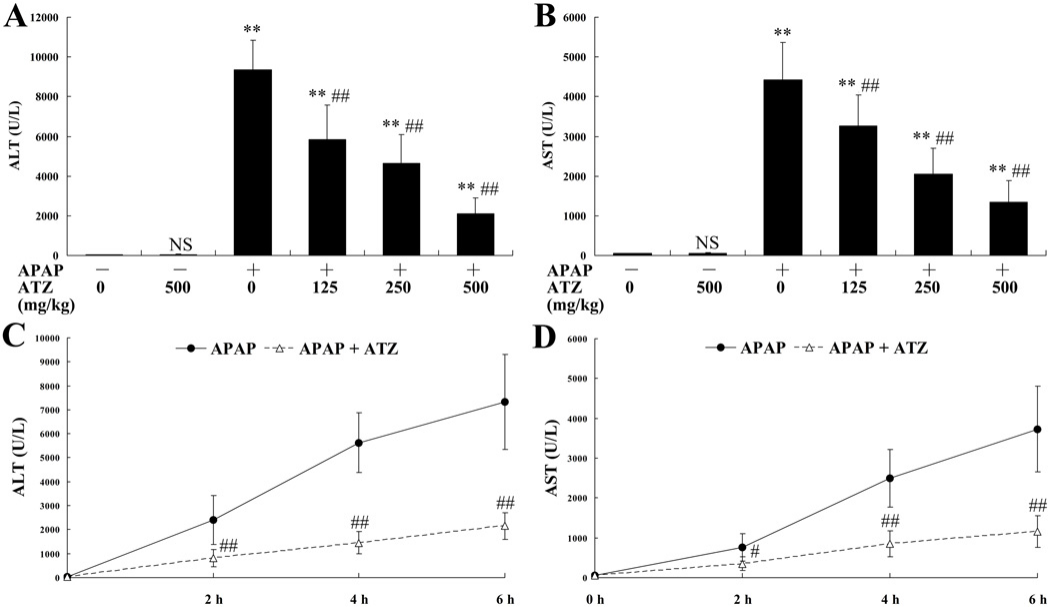
Pretreatment with ATZ suppressed the elevation of plasma aminotransferases induced by APAP. Mice were pretreated with vehicle or indicated doses of ATZ before APAP exposure. The plasma levels of alanine aminotransferase (ALT, A) and aspartate aminotransferase (AST, B) were determined at 8 h after APAP exposure. Data were expressed as mean ± SD, n = 8. * *P*<0.05, ** *P*<0.01, as compared with APAP group (APAP +/ATZ 0). In another set of animals, the mice were treated with either vehicle or ATZ (500 mg/kg) before APAP exposure. The mice were sacrificed at 2 h, 4 h or 6 h after APAP exposure and the plasma levels of ALT (C) and AST (D) were determined. The data were expressed as the mean ± SD, n = 8. ^NS^ *P*>0.05, ** *P*<0.01, as compared with the CON group; ^#^ *P*<0.05, ^##^ *P*<0.01, as compared with the APAP group.

**Fig 2:**
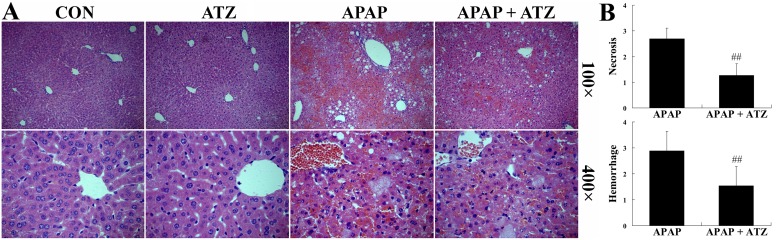
Pretreatment with ATZ alleviated liver histological abnormalities induced by APAP. Liver samples were harvested at 8 h after APAP exposure and the liver sections were stained with hematoxylin-eosin for morphological evaluation. (A) The representative liver sections of each group are shown. (B) The degree of hepatocellular necrosis and hemorrhage were semi-quantified using a 0 (no lesion) to 4 (severe change) scoring system in 20 random fields at 100× magnification per animal (n = 3 per group). Data were expressed as mean ± SD, ^##^ *P*<0.01, as compared with the APAP group.

**Fig 3:**
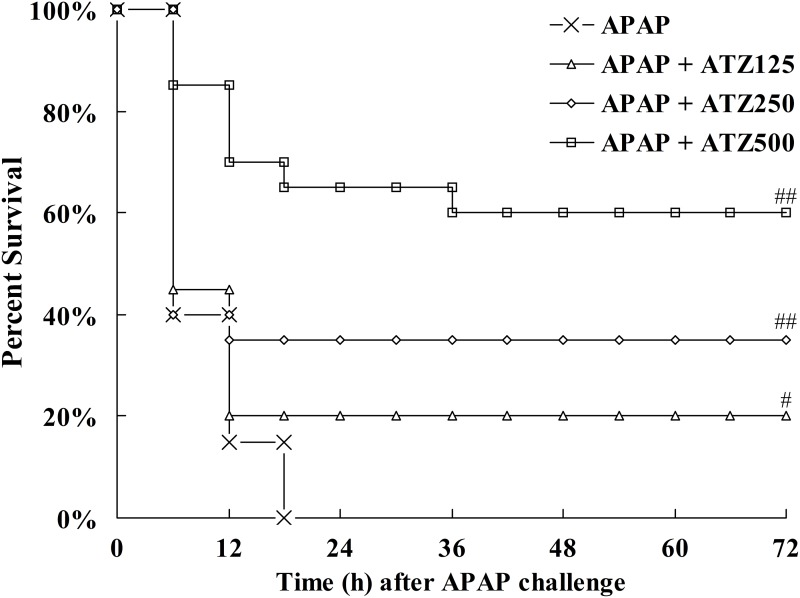
Pretreatment with ATZ decreased the mortality induced by APAP. Mice were pretreated with vehicle or various doses of ATZ (125 mg/kg, 250 mg/kg, 500 mg/kg) before APAP exposure. Survival was monitored and the percent survival rate was expressed as Kaplan-Meier survival curves (n = 20). ^#^ *P*<0.05, ^##^ *P*<0.01, as compared with the APAP group.

The levels of ALT and AST, widely used as quantitative biochemical markers of hepatocellular damage [23], increased significantly after APAP challenge, but the elevation of these aminotransferases was suppressed in mice pretreated with ATZ in a dose and time-dependent manner (Fig 1). The histological examination found that intraperitoneal injection of APAP resulted in obvious histological abnormalities, including massive cell necrosis and extensive hemorrhage in liver tissues. Consistent with the decreased elevation of aminotransferases, pretreatment with ATZ markedly attenuated APAP-induced histological lesions (Fig 2). Our data also showed that pretreatment with ATZ dose-dependently improved the survival rate of APAP-challenged mice (Fig 3). In addition, the plasma aminotransferases level (Fig 1), the hepatic histological architecture (Fig 2) and the survival rate (data not shown) in mice treated with ATZ alone were not different from those of the control mice.

### Pretreatment with ATZ suppressed APAP-induced oxidative stress

**Fig 4:**
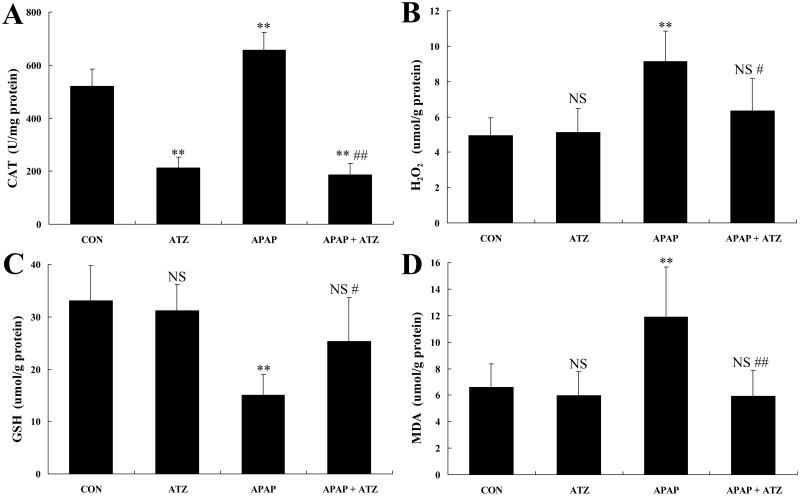
Pretreatment with ATZ suppressed CAT activities, H_2_O_2_ contents, GSH contents and MDA contents in the liver of APAP exposed mice. Hepatic CAT activities (A), H_2_O_2_ contents (B), GSH contents (C) and MDA contents (D) were determined at 8 h after APAP exposure. Data were expressed as mean ± SD, n = 8. ^NS^ *P*>0.05, ** *P*<0.01, as compared with the CON group; ^#^ *P*<0.05, ^##^ *P*<0.01, as compared with APAP group.

ATZ is a widely used CAT inhibitor [9–10], the intraperitoneal administration of ATZ significantly suppressed the activities of CAT in mice with or without APAP exposure (Fig 4A). CAT is the major antioxidant enzyme that catalyzes the decomposition of H_2_O_2_ [8]. Although ATZ significantly suppressed hepatic CAT activity, we unexpectedly found that ATZ reduced H_2_O_2_ levels in liver tissue of APAP-challenged mice (Fig 4B). In APAP exposed mice, hepatic contents of reduced glutathione decreased significantly, while ATZ partially reversed this abnormality (Fig 4C). The level of MDA, an end product of lipid peroxidation in liver damage [24], was determined to evaluate the degree of oxidative injury. As shown in Fig 4D, APAP exposure induced significant increase of MDA, but ATZ markedly decreased the MDA contents in APAP exposed mice.

### Pretreatment with ATZ suppressed APAP-induced inflammatory response

**Fig 5:**
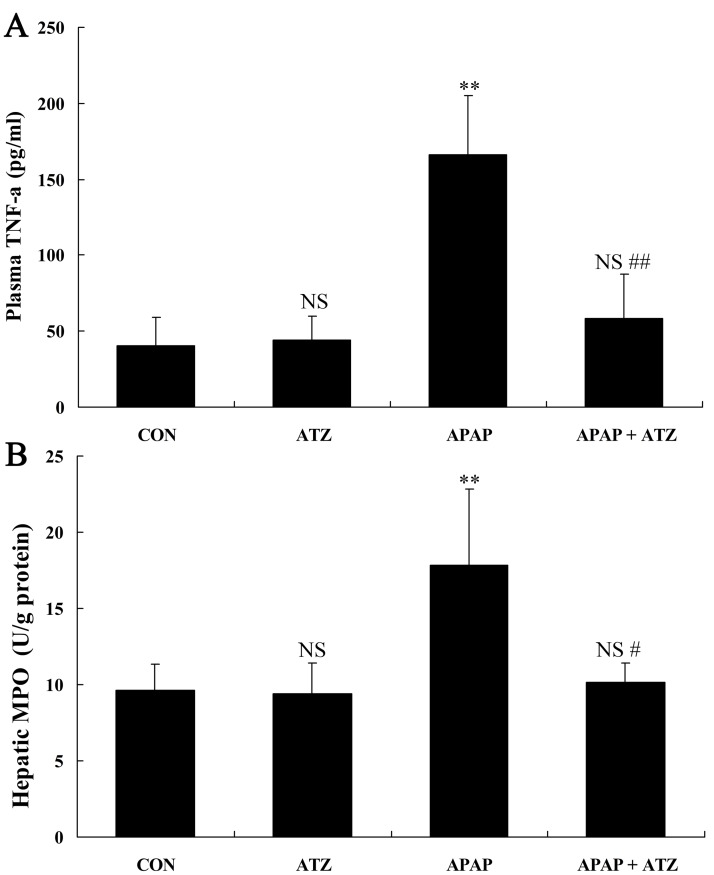
Pretreatment with APAP suppressed the levels of plasma TNF-α and hepatic MPO in APAP exposed mice. Plasma TNF-α levels (A) and hepatic MPO activities (B) were determined at 8 h after APAP exposure. Data were expressed as mean ± SD, n = 8. ^NS^ *P*>0.05, ** *P*<0.01, as compared with the CON group; ^#^ *P*<0.05, ^##^ *P*<0.01, as compared with APAP group.

TNF-α is an important proinflammatory cytokine involved in progression of APAP-induced hepatotoxicity [25–26]. In APAP exposed mice, the plasma levels of TNF-α increased significantly whereas the levels of TNF-α in APAP-challenged mice were suppressed by ATZ (Fig 5A). The infiltration of neutrophils was quantified by the MPO activity in liver tissues [27]. As expected, MPO activities increased significantly in APAP-challenged mice. In mice treated with ATZ, the upregulation of MPO by APAP was obviously lower (Fig 5B).

### Pretreatment with ATZ decreased JNK activation

**Fig 6:**
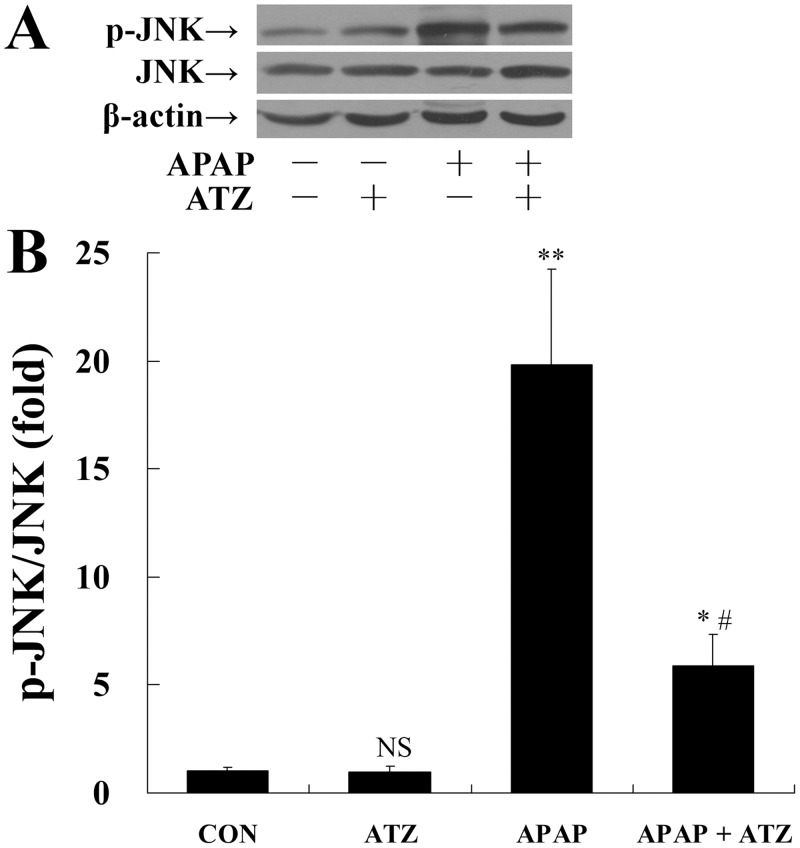
Pretreatment with ATZ decreased the level of phosphorylated JNK in APAP exposed mice. Mice were treated with vehicle or ATZ (500 mg/kg) at 30 min before APAP exposure. Liver samples were harvested at 8 h after APAP exposure. The levels of phosphorylated JNK and total JNK were determined by Western blot analysis. (A) The bands of phosphorylated JNK, total JNK and β-actin were indicated by arrows. (B) Western blots were scanned by densitometry and data presented as relative intensity units. Data were expressed as mean ± SD, n = 8. ^NS^ *P*>0.05, * *P*<0.05, ** *P*<0.01, as compared with the CON group; ^#^ *P*<0.05, as compared with APAP group.

The phosphorylation and activation of JNK is a crucial molecular event in the pathogenesis of APAP hepatotoxicity [21]. Western blot analysis indicated that APAP challenge markedly increased the phosphorylation level of JNK in the liver, while ATZ suppressed the level of phosphorylated JNK (Fig 6).

### Pretreatment with ATZ downregulated CYP2E1 expression

**Fig 7:**
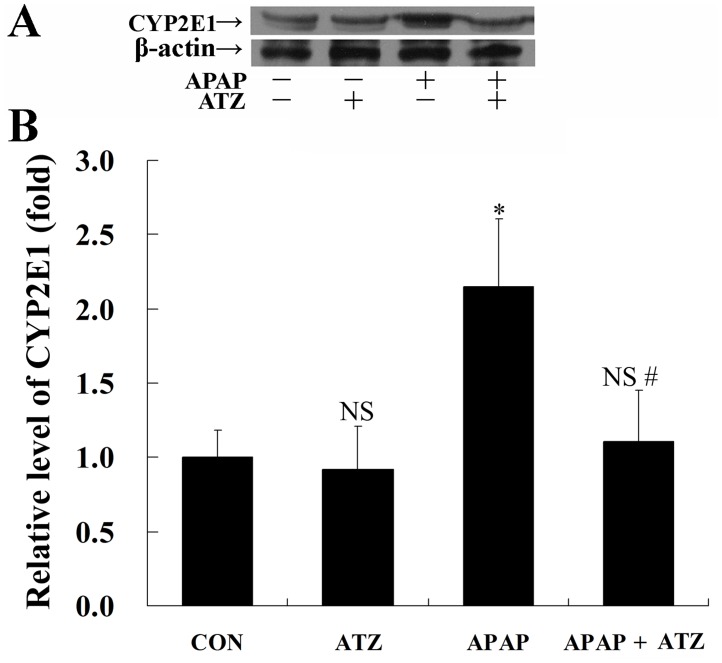
Pretreatment with ATZ decreased the level of CYP2E1 in APAP exposed mice. Mice were treated with vehicle or ATZ (500 mg/kg) at 30 min before APAP exposure. Liver samples were harvested at 8 h after APAP exposure. The levels of CYP2E1 were determined by Western blot analysis. (A) The bands of CYP2E1 and β-actin were indicated by arrows. (B) Western blots were scanned by densitometry and data presented as relative intensity units. Data were expressed as mean ± SD, n = 8. ^NS^ *P*>0.05, * *P*<0.05, as compared with the CON group; ^#^ *P*<0.05, as compared with the APAP group.

APAP is metabolically activated mainly by CYP2E1 [28]. The present study showed that APAP-challenge markedly increased the contents of hepatic CYP2E1 protein. Treatment with ATZ significantly suppressed the induction of CYP2E1 (Fig 7).

### Posttreatment with ATZ also attenuated APAP induced liver injury

**Fig 8:**
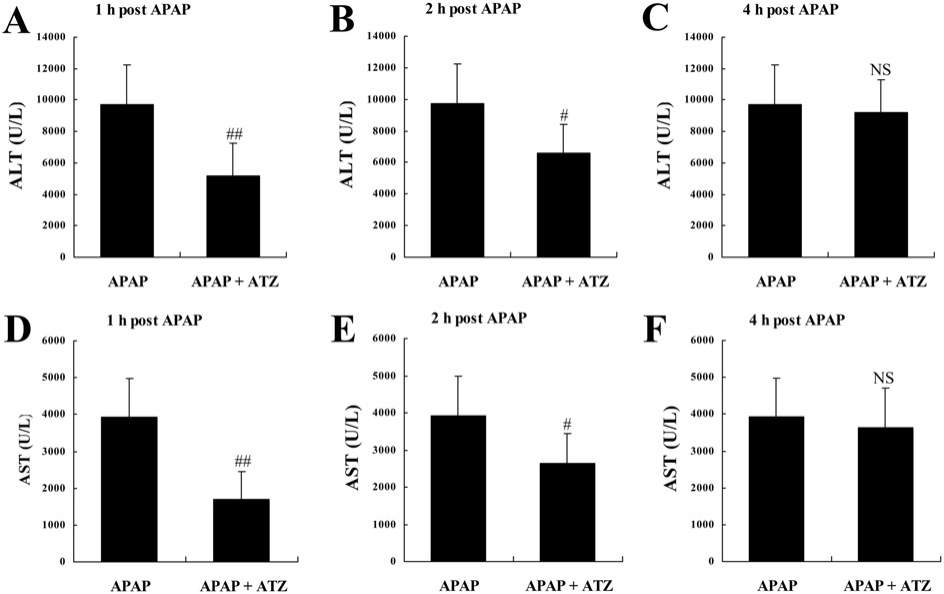
Posttreatment with ATZ suppressed the elevation of plasma aminotransferases induced by APAP. Mice were treated with vehicle or ATZ (500 mg/kg) at 1 h (A and D), 2 h (B and E) or 4 h (C and F) after APAP exposure. The plasma levels of alanine aminotransferase (ALT, A-C) and aspartate aminotransferase (AST, D-F) were determined at 8 h after LPS/D-Gal exposure. Data were expressed as mean ± SD, n = 8. ^NS^ *P*>0.05, ^#^ *P*<0.05, ^##^ *P*<0.01, as compared with the APAP group.

**Fig 9:**
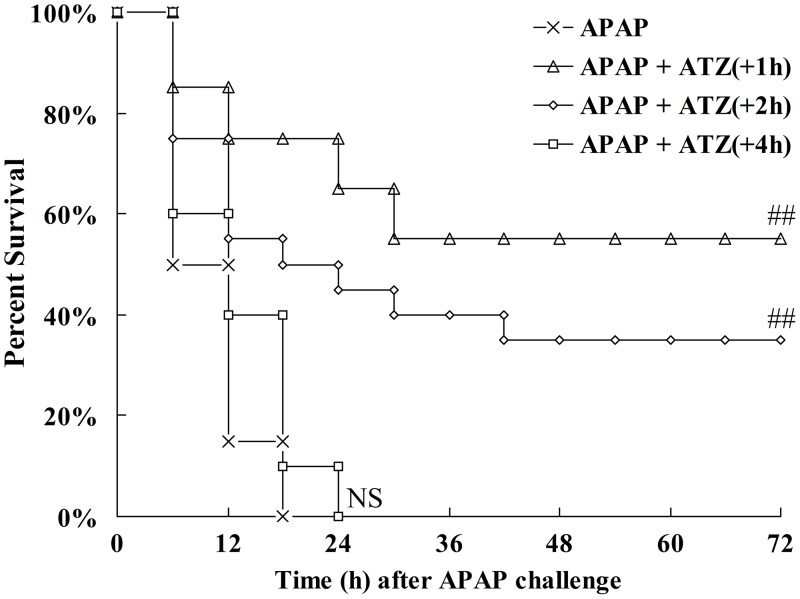
Posttreatment with ATZ decreased the mortality induced by APAP. Mice were treated with vehicle or ATZ (500 mg/kg) at 1 h, 2 h or 4 h after APAP exposure. Survival was monitored and the percent survival rate was expressed as Kaplan-Meier survival curves (n = 20). ^NS^ *P*>0.05, ^##^ *P*<0.01, as compared with the APAP group.

**Fig 10:**
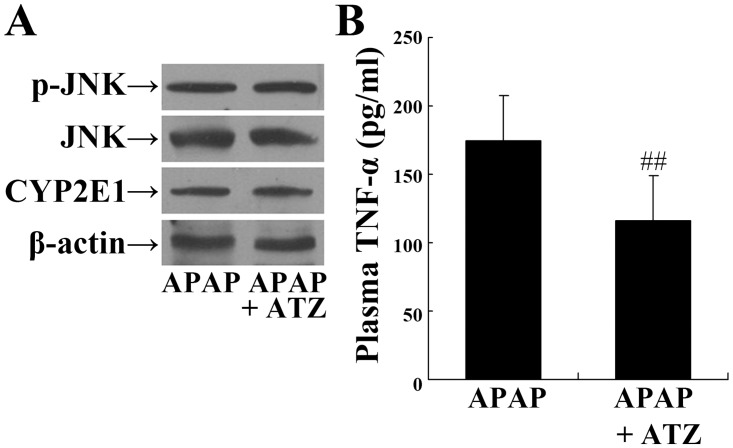
Posttreatment with ATZ suppressed the levels of plasma TNF-α but had no effects on JNK phosphorylation or CYP2E1 expression in APAP exposed mice. Mice were treated with vehicle or ATZ (500 mg/kg) at 1 h after APAP exposure. Hepatic levels of phosphorylated JNK, total JNK, CYP2E1, β-actin (A) and plasma levels of TNF-α (B) were determined at 8 h after APAP exposure. Data were expressed as mean ± SD, n = 8. ^##^ *P*<0.01, as compared with the APAP group.

Since the pre-insult treatment protocol is different from clinic settings and seems unrealistic in clinical treatment, the pharmacological efficiency of ATZ treated with clinically relevant post-insult administration strategy was also tested. The experimental data indicated that ATZ administered at 1 h or 2 h after APAP challenged could also decrease the elevation of ALT and AST. However, treatment with ATZ at 4 h after APAP exposure had no obvious effect on the levels of ALT and AST (Fig 8A–8F). In addition, the administration of ATZ at 1 h or 2 h after APAP exposure significantly improved the survival rate of mice (Fig 9). The data also indicated that posttreatment with ATZ significantly suppressed APAP-induced production of TNF-α but it had no effects on the levels of phosphorylated JNK or CYP2E1 (Fig 10).

### The safety of ATZ in mice

Finally, the LD_50_ and the 95% confidence limits of ATZ were determined to evaluate the safety of ATZ treatment. Our data indicated that the LD_50_ of ATZ in mice was 5367.4 mg/kg body weight and the 95% confidence limits of ATZ were 5260.4–5476.6 mg/kg (Table 1), which is much higher then the dose of ATZ in the present study.

**Table 1:** The LD_50_ and the 95% confidence limits of ATZ in mice (n = 20).

Dose (mg/kg)	Mortality rate (%)	LD_50_ (mg/kg)	95%confidence interval (mg/kg)
5200	20		
5460	60	5367.4	5260.4–5476.6
5733	100		
6019	100		

## Discussion

APAP overdose is the most common cause of drug-induced liver injury in developed countries [2]. The severe oxidative stress induced by the highly reactive metabolite NAPQI plays central roles in the pathogenesis of APAP-induced hepatotoxicity [20]. The endogenous antioxidant enzymes CAT usually provides protective benefits in oxidative stress [8]. However, the present study found that the widely used CAT inhibitor ATZ significantly protected mice from APAP-induced acute liver injury. The protective benefits were evidenced by suppressed elevation of plasma aminotransferases, improved histopathological abnormality and increased survival rate.

It is well known that the CAT plays defensive roles in oxidative stress via catalyzing the decomposition of H_2_O_2_ [8]. Inhibition of CAT by ATZ resulted in enhanced the cytotoxicity of Alzheimer’s amyloid-beta peptide in both neuronal and non-neuronal cells [13], ATZ treatment also resulted in significantly higher elevations in intracellular free radicals and significantly lowered cell viability of fibroblasts exposed to UV radiation [14]. But in the present study, as well as in our previous study, suppression of CAT by ATZ decreased the levels of H_2_O_2_, reduced the generation of MDA and attenuated APAP or CCl_4_-induced hepatotoxicity [16]. The decreased levels of H_2_O_2_ and MDA in these studies could not be explained by the suppressed activity of CAT. Although there was no direct evidence indicating that CAT could provide beneficial effects in APAP poisoning, the crucial protective function of CAT in CCl_4_-induced hepatotoxicity was well proven in acatalasemic mice and transgenic mice [17, 29]. Therefore, the hepatoprotective effects of ATZ in APAP or CCl_4_-induced hepatotoxicity might not be attributed to its inhibitory effects on CAT.

The level of H_2_O_2_ is determined by both the biochemical generation and its degradative clearance. In the present study, inhibition of CAT by ATZ might block the degradation of H_2_O_2_, but these were not accompanied with increase in H_2_O_2_. Therefore, we questioned whether ATZ could modulate other target that involved in the metabolism of APAP and the generation of H_2_O_2_. The CYP2E1 is the major enzyme catalyzing the metabolism of APAP to hepatotoxic reactive metabolite [30]. The expression of CYP2E1 was induced by APAP and other hepatotoxic substance such as alcohol [28, 31–32]. The induction of CYP2E1 was implicated in alcohol-mediated increases in APAP hepatotoxicity [33], whereas suppression of CYP2E1 expression by tea polyphenols and other compounds attenuated APAP hepatotoxicity [34–35]. In the present study, the induction of CYP2E1 in APAP exposed mice was inhibited by pretreatment with ATZ, which might lead to suppressed generation of reactive metabolite and alleviated liver damage. Interestingly, the hepatotoxicity of CCl_4_ also largely depends on CYP2E1, CCl_4_ is mainly metabolized by CYP2E1 in the hepatocytes to produce the highly reactive free radicals, which lead to cellular damage [36]. Therefore, downregulation of CYP2E1 also seems to be a plausible explanation for the protective effects of ATZ on CCl_4_ hepatotoxicity.

There is increasing evidence that the JNK plays a pivotal role in the induction of APAP hepatotoxicity [20]. The JNK is sustained phosphorylated and activated in human and murine liver with APAP-induced hepatotoxicity [37]. The activated JNK translocates to mitochondria, inducing mitochondria permeability transition and triggering hepatocyte death [38]. Inhibition of JNK by specific JNK inhibitors effectively rescued mice from APAP-induced liver injury [37]. It was reported that JNK was sensitive to APAP-induced generation of reactive oxygen species such as H_2_O_2_ [21, 39]. In the present study, the induced phosphorylation of JNK in APAP-exposed mice could be suppressed by pretreatment with ATZ, which was correlated well with the downregulated CYP2E1 and the alleviated liver injury.

More importantly, ATZ treated with clinically relevant post-insult administration strategy at the early stage of APAP exposure also decreased the levels of plasma aminotransferases and increased the survival rate APAP-challenged mice. However, the alleviated liver injury by ATZ posttreatment was not associated with CYP2E1 downregulation and JNK inhibition. Therefore, other CYP2E1-independent mechanisms might also be involved in the hepatoprotective effects of ATZ. In addition to oxidative stress induced tissue injury, the recruitment of neutrophils and the production of proinflammatory cytokines are critical for the progression of APAP hepatotoxicity [40–41]. In the present study, pretreatment with ATZ significantly suppressed APAP-induced elevation of hepatic MPO and the production of TNF-α. The plasma level of TNF-α in APAP-exposed mice also decreased in ATZ-posttreated group. The anti-inflammatory effects of ATZ have been confirmed in LPS-stimulated neutrophils in vitro and LPS-induced acute lung injury in vivo [15]. Moreover, TNF-α is a representative proinflammatory cytokine acting as a detrimental factor in various liver disorders and the presence of TNF-α significantly enhanced the toxicity of APAP to hepatocytes [25, 42–43]. These data suggested that the sterile inflammation in APAP challenged mice was inhibited after ATZ treatment. The anti-inflammatory action of ATZ might be another beneficial mechanism underlying the hepatoprotective effects of ATZ in APAP poisoning.

Taken together, the present study has shown that ATZ effectively attenuated liver damage and decreased mortality induced by APAP poisoning. The downregulation of CYP2E1 induction and the suppression of inflammation might be the important mechanisms underlying these protective benefits of ATZ. Importantly, treatment with ATZ with clinically relevant post-insult administration strategy could also provide therapeutic benefits and the therapeutic dose of ATZ is much lower than its LD_50_. Therefore, ATZ seems effective and safe in protecting mice against APAP-induced hepatotoxicity, but the underlying mechanisms remain to be further investigated.
